# Dual-Wavelength Volumetric Microlithography for Rapid Production of 4D Microstructures

**DOI:** 10.1021/acsami.4c01883

**Published:** 2024-04-22

**Authors:** Alexandra Gruzdenko, Dirk J. Mulder, Albert P. H. J. Schenning, Jaap M. J. den Toonder, Michael G. Debije

**Affiliations:** † Stimuli-Responsive Functional Materials and Devices, Department of Chemical Engineering and Chemistry, Eindhoven University of Technology, P.O. Box 513, 5600 MB Eindhoven, The Netherlands; ‡ Microsystems, Department of Mechanical Engineering, Eindhoven University of Technology, P.O. Box 513, 5600 MB Eindhoven, The Netherlands; § Interactive Polymer Materials (IPM), Eindhoven University of Technology, Groene Loper 3, 5612 AE Eindhoven, The Netherlands; ∥ Institute for Complex Molecular Systems, Eindhoven University of Technology, Den Dolech 2, 5600 MB Eindhoven, The Netherlands; ⊥ Photosynthetic, De Boelelaan 1085, 1081HV Amsterdam, The Netherlands

**Keywords:** dual-wavelength volumetric microlithography, liquid crystals, 4D printing, microactuators, responsive microstructures

## Abstract

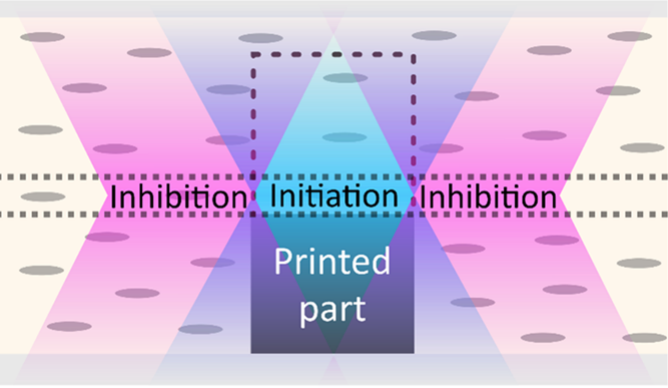

4D microstructured actuators are micro-objects made of stimuli-responsive materials capable of induced shape deformations, with applications ranging from microrobotics to smart micropatterned haptic surfaces. The novel technology dual-wavelength volumetric microlithography (DWVML) realizes rapid printing of high-resolution 3D microstructures and so has the potential to pave the way to feasible manufacturing of 4D microdevices. In this work, DWVML is applied for the first time to printing stimuli-responsive materials, namely, liquid crystal networks (LCNs). An LCN photoresist is developed and characterized, and large arrays of up to 5625 LCN micropillars with programmable shape changes are produced by means of DWVML in the time span of seconds, over areas as large as ∼5.4 mm^2^. The production rate of 0.24 mm^3^ h^–1^ is achieved, exceeding speeds previously reported for additive manufacturing of LCNs by 2 orders of magnitude. Finally, a membrane with tunable, micrometer-sized pores is fabricated to illustrate the potential DWVML holds for real-world applications.

## Introduction

Researchers have worked for decades to produce high-resolution, three-dimensional (3D) structures at the microscale. Many techniques, including two-photon polymerization-direct laser writing (TPP-DLW), stereolithography (SLA), and digital light processing (DLP), have been established^1^ and yet more emerge (xolography (XG),^2^ computed axial lithography (CAL),^3^ flow lithography^4^), offering different resolution, shape complexity, and production speeds.^5^ However, in most cases, static 3D microstructures are produced. Stimuli-responsive polymer materials like hydrogels, magnetic polymers, and liquid crystal (LC) networks (LCNs) and elastomers (LCEs) (jointly termed liquid crystal polymers (LCPs)) capable of externally triggered shape deformations can be utilized to make 3D micro-objects that change their configuration in time.^6^ Many applications of such systems, commonly termed 4D, are envisioned, including targeted drug delivery, cell manipulation, microsurgery,^7,8^ smart surfaces for haptic technologies or controlled wettability,^9^ microrobotics,^10,11^ microassembly,^12,13^ photonics,^14−16^ and advanced microfluidics.^17^

LCPs^18,19^ are particularly attractive for producing 4D microstructures since they exhibit reversible, programmable, and complex shape changes, based on the disruption of the orientational order of the anisotropically shaped LC molecules. LCPs can be made responsive to various stimuli including temperature, light, chemical composition, and electric and magnetic fields, and can operate both in dry and aqueous environments. Microfabrication techniques that have been applied to these materials to date include TPP-DLW,^20−22^ soft lithography (SL),^23,24^ photolithography (PL),^11^ dispersion polymerization (DP),^25^ and microfluidics (MF).^26−29^ While finding applications in research, these approaches have limitations. SL, DP, and MF offer only limited control over LC alignment, and SL, PL, DP, and MF cannot produce complex or truly 3D shapes. TPP-DLW allows for unprecedent resolution and shape complexity and good LC alignment control but is relatively slow (with printing speeds of <0.01 mm^3^ h^–1^ reported for LCP resists^21,30^) since structures are printed point by point. Therefore, it may not be suitable for large-scale manufacturing of microdevices.

A new technology called dual-wavelength volumetric microlithography (DWVML) has been recently developed^31^ that makes use of digital light processing and microscopy optics to rapidly print (up to 100 mm^3^ h^–1^) 2D, 2.5D, and high-aspect ratio 3D microstructures layer by layer, over large surface areas, with resolution down to 100 nm, thanks to the employment of localized photoinduced initiation and inhibition. Importantly for LCPs, in contrast to other additive manufacturing methods such as SLA, XG, CAL, and alternative DLP-based approaches,^32−35^ many conventional LC alignment methods are expected to be compatible with DWVML since fabrication is done not in vats but the same cells widely used in liquid crystal research.

DWVML has never been applied to 4D microfabrication. While its good balance between resolution, speed, and shape complexity makes it very attractive for printing a wide variety of stimuli-responsive materials, we now report using DWVML to rapidly produce microstructures based on LCPs for which the technique is particularly promising. The basic principles of the technology are introduced, and a liquid crystal photoresist suitable for DWVML-processing is developed. Then, arrays of up to several thousand LCN micropillars with different alignments are fabricated in tens of seconds over areas as large as ∼5.4 mm^2^. Finally, a membrane with tunable micrometer-sized pores is printed as a simple example of a potential application.

## Results and Discussion

### Basic Principle Of Dual-Wavelength Volumetric Microlithography

**Figure 1:**
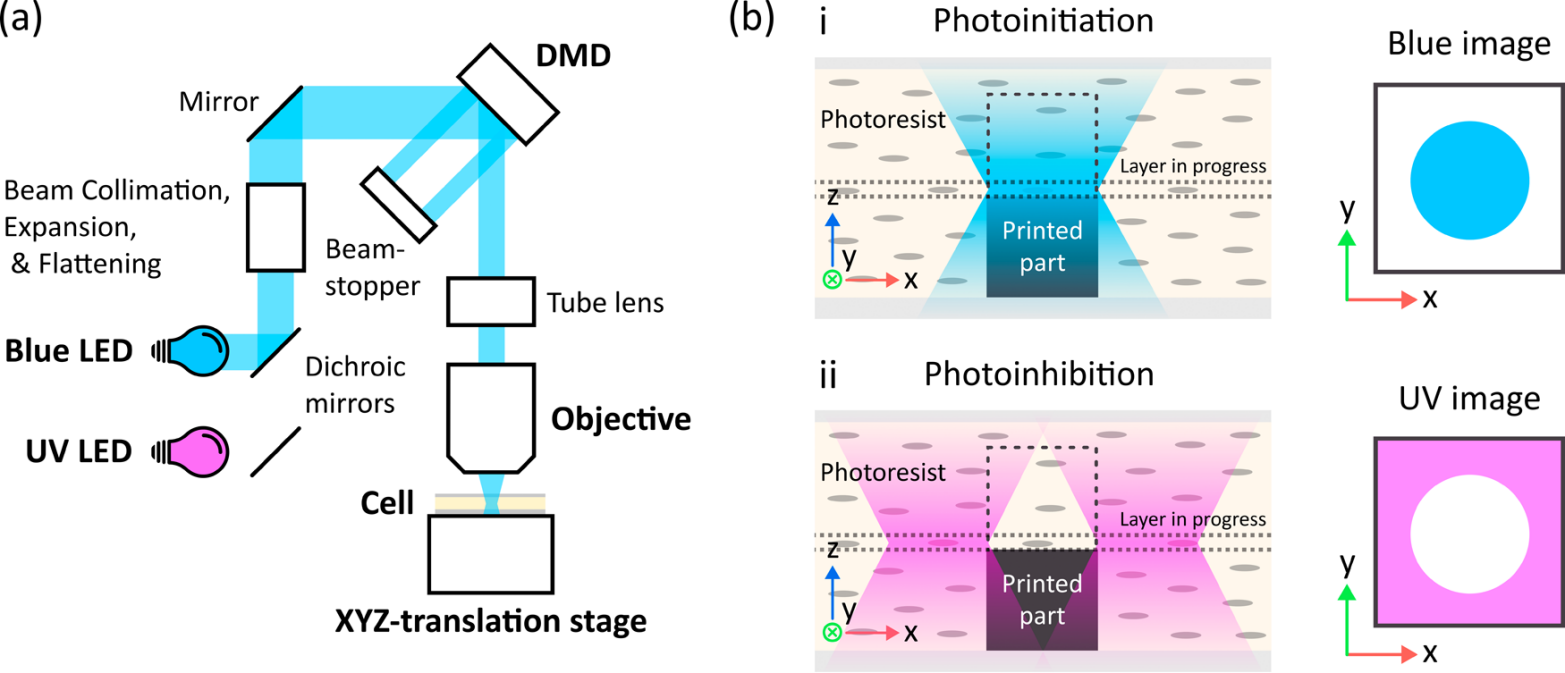
(a) Schematic of the DWVML setup. (b) DWVML-printing process: (i) a blue image (right) of a cross section of a microstructure’s layer (the case of a pillar is shown) is projected into a cell filled with photoresist, e.g., liquid crystals containing a blue light-responsive photoinitiator (left); (ii) in alternation with the blue image, UV is projected around the layer in progress (right) to activate a UV-responsive inhibitor and confine polymerization to the desired area (left).

DWVML-printing is performed in a cell consisting of two glass substrates sandwiched together, with the distance between them controlled by microspacers. The cell is filled with a photoresist containing monomers, blue light-activatable photoinitiator camphorquinone (CQ), and tertiary amine co-initiator EDAB (both required for type-II photoinitiation). To realize layer-by-layer printing, light from a 455 nm blue LED is directed to a digital micromirror device (DMD) that generates images of planar cross sections of layers into which a desired shape is divided (Figure 1a,b(i)). A microscope objective reduces the images in size and projects them one by one at the required depths in the cell, the latter being controlled by the position of a translation stage holding the sample. Printing starts at the bottom glass slide with the lowest cross section. A coverslip is utilized as the top cell substrate to minimize optical aberrations.

To prevent loss of resolution caused by factors including radical diffusion, light scattering into nontarget areas, and polymerization outside of the focal plane due to the overlap of DMD-generated beams (all effects intrinsic to photopolymerization-based microfabrication methods in general^36,37^), an inhibition-patterning technique^38,39^ is used to confine the polymerization reaction to a desired area. This process involves projecting 385 nm ultraviolet (UV) light around the layer in progress to activate the UV responsive compound *o*-Cl-HABI that is also present in the photoresist and acts as an inhibitor (Figure 1b(ii)). The blue and UV images are projected alternately at a high frequency. The interplay between the two illumination wavelengths defines the final regions of polymerization and inhibition in the build volume.

Once printing is completed, the cell is placed in a developer solution for 15 min and then opened to wash away nonpolymerized material; the printed microstructure remains on the glass substrate. A comprehensive discussion of the technology can be found elsewhere.^31^

### Liquid Crystal Photoresist

**Figure 2:**
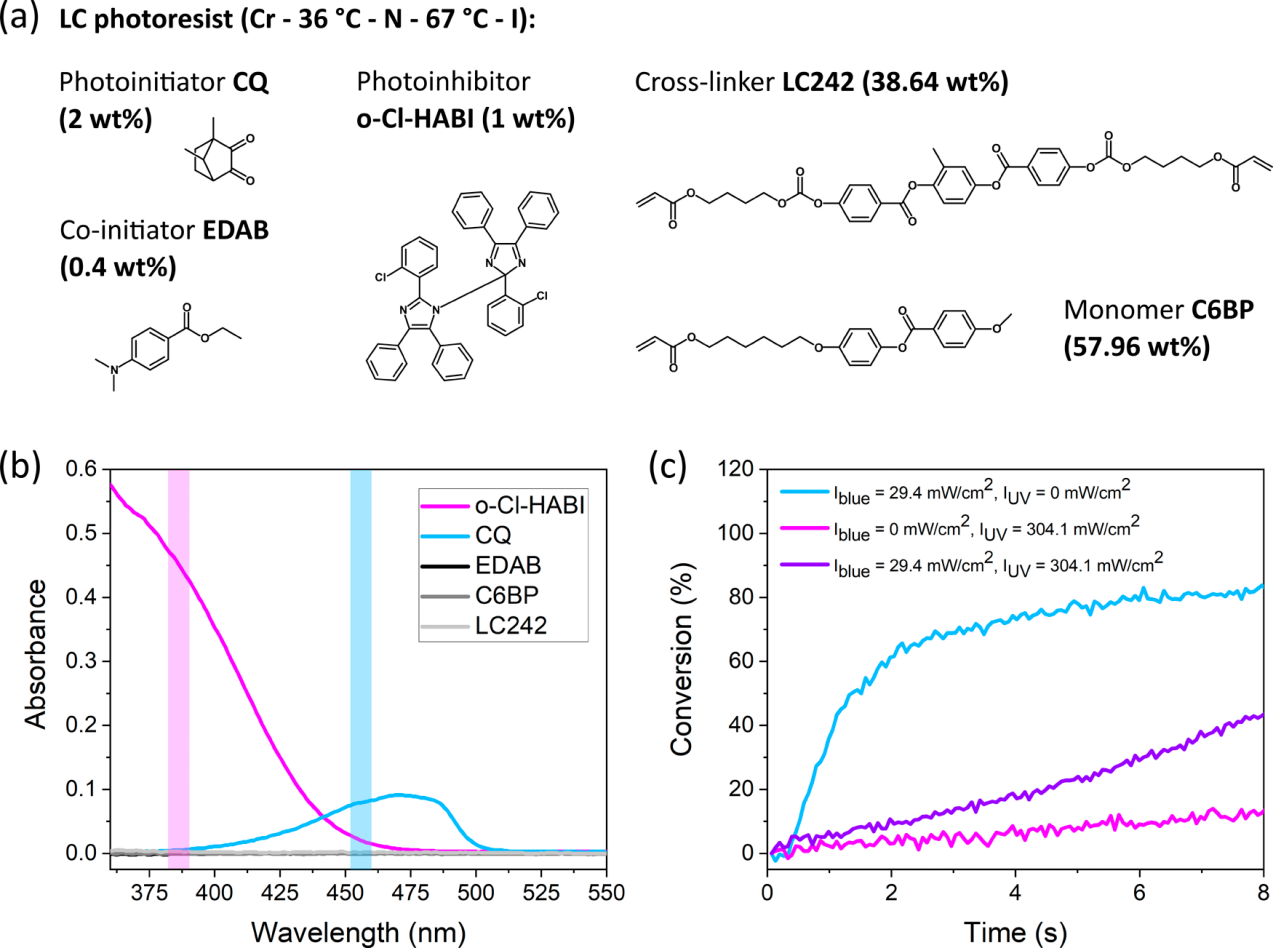
(a) Chemical composition of the LC photoresist. (b) Absorbance spectra of the LC photoresist components measured for 2.5 mM solutions in THF. The blue and magenta bars indicate the initiating and inhibiting wavelengths used in the DWVML setup, namely 455 and 385 nm, respectively. (c) Acrylate conversion as a function of time measured for the LC photoresist when continuously illuminated with blue (455 nm), UV (385 nm), and both blue and UV lights simultaneously at the estimated optimized intensities for rapid production of robust microstructures. Corresponding light intensities are indicated in the graph.

An LC photoresist for DWVML was prepared from LC monomer C6BP (∼57.96 wt %), LC cross-linker LC242 (∼38.64 wt %), photoinitiator CQ (∼2 wt %), co-initiator EDAB (∼0.4 wt %), and photoinhibitor o-Cl-HABI (∼1 wt %) (Figure 2a). Owing to the supercooling behavior of the LC monomer and cross-linker, the resist, with *T*_CrN_ ≈ 36 °C and *T*_NI_ ≈ 67 °C (Figure S1a), remained in the nematic state for ∼1.5 h after cooling to 25 °C, allowing sufficient time for DWVML-processing at room temperature (RT). The resulting polymerized material experienced the glass transition at *T*_g_ ≈ 27 °C and the nematic–isotropic transition at *T*_NI_ ≈ 210 °C (Figure S1b).

The absence of spectral overlap between the photoinitiator and photoinhibitor, crucial for confining photopolymerization in DWVML, was confirmed by observing the near-exclusive absorption at 455 nm by CQ and at 385 nm by *o*-Cl-HABI (Figure 2b). Additionally, no absorbance at the two wavelengths was observed for the other resist components, suggesting that they should not interfere with the activation of the photoagents.

To identify optimal illumination conditions for DWVML-printing, polymerization kinetics of the LC photoresist under exposure to only blue, only UV, and both blue and UV light simultaneously were characterized using real-time Fourier transform infrared (FTIR) spectroscopy. The acrylate C=C bond peak (1635 cm^–1^) was monitored in a thin (5–10 μm) layer of the nematic LC material sandwiched between an ATR crystal and a coverslip (see Experimental Section for more details). The results for light intensities allowing for an optimal photoresponse are presented in Figure 2c. Blue light (29.4 mW cm^–2^) initiated rapid polymerization via excitation of CQ (reaching ∼80% acrylate conversion within 8 s) while shining both blue (29.4 mW cm^–2^) and UV (304.1 mW cm^–2^) lights resulted in the notable reduction of the polymerization rate. Exposure to exclusively UV light (304.1 mW cm^–2^) induced only minor conversion (<15%) in the studied time range (the presence of initiator-less UV-induced polymerization typical for some LC monomers^40^ was tested but not observed for C6BP and LC242 employed in this study (see SI)).

An optimized overall exposure time of ∼1 s was chosen for printing since in the areas with only UV or both UV and blue light present, polymerization was limited; while in regions exposed to only blue light, sufficient monomer conversion was achieved to rapidly produce robust LCN microstructures (see below).

### DWVML-Fabricated LCN Microactuators

To characterize the print quality and production speed, DWVML was used to print a 5 × 5 array of micropillars with circular cross sections. Adhesive and antiadhesive layers were spin-coated on the bottom and top cell substrates, respectively, to avoid loss of microstructures during the washing step and ensure that all prints remained on the same glass slide. The cell was filled with the LC resist at 80 °C (in the isotropic phase) by capillary action and then cooled to RT. The structures were then printed from the bottom to the top cell substrate layer by layer. The translation stage was moved continuously at a speed of 14.4 nm ms^–1^. The projected image changed every 5.8 ms, resulting in an effective layer thickness of ∼200 nm (see Experimental Section). To facilitate rapid microfabrication, the pillars were printed simultaneously by using blue and UV images encompassing all 25 structures. 29.4 mW cm^–2^ blue and 304.1 mW cm^–2^ UV lights were used to initiate and inhibit polymerization, respectively. The whole printing process took 1.2 s.

**Figure 3:**
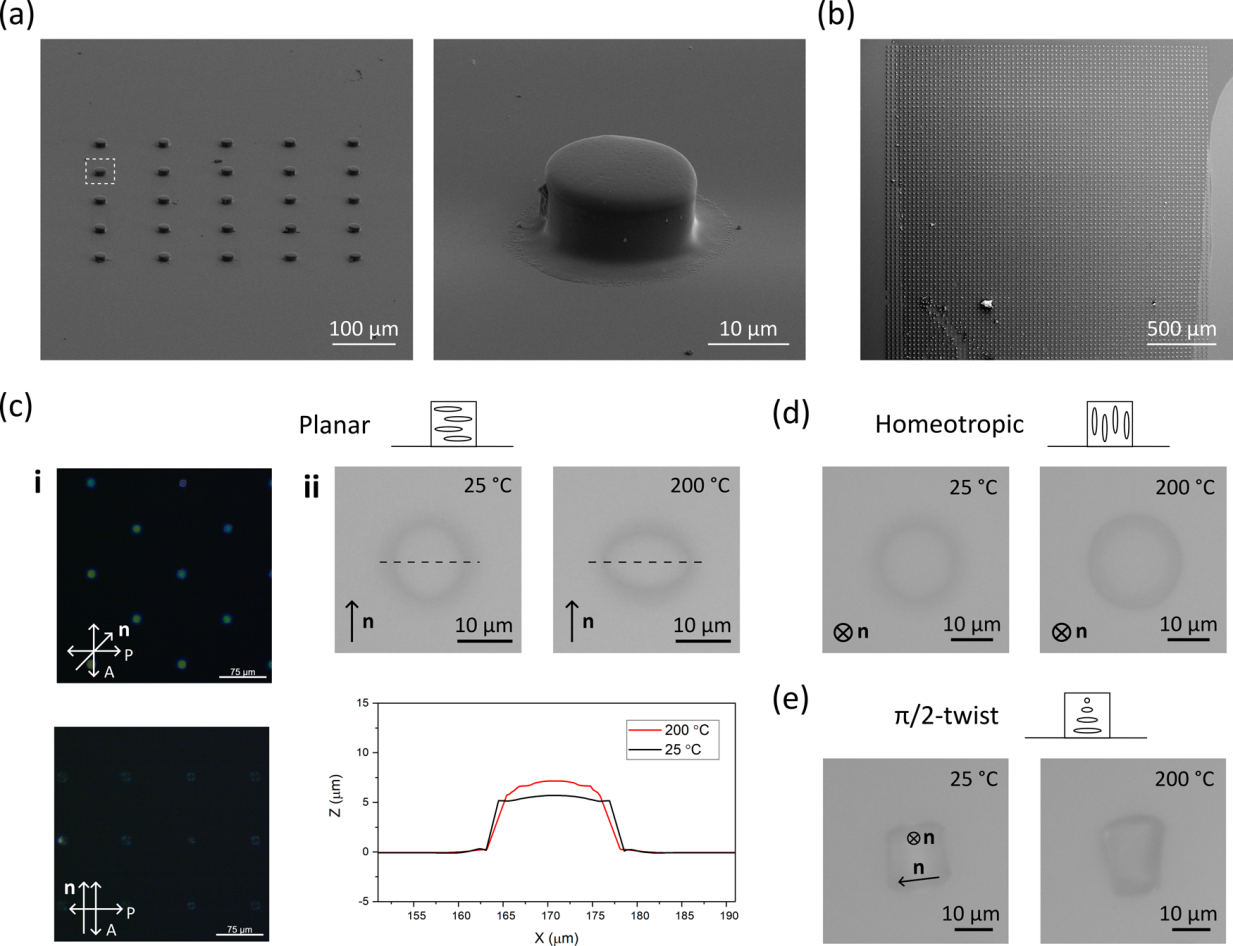
DWVML-printed micropillars. (a) SEM image of a 5 × 5 pillar array printed in 1.2 s (left) and a close-up of one of the pillars (right). (b) SEM image of an array of 75 × 75 = 5625 pillars produced in 37.5 s by printing subarrays of 15 × 15 microcylinders adjacent to each other. (c) Pillars printed in a planar cell: (i) POM images; (ii) top-view reflection images and profile measurements illustrating actuation of one of the planar pillars. The profiles were measured along the dashed lines shown in the reflection images. (d, e) Top-view reflection images illustrate actuation of homeotropic and π/2-twist pillars.

In Figure 3a, the resulting array of 25 microstructures is shown. The pillars had the intended circular shape and were uniformly sized with an average diameter of 19.0 ± 0.8 μm and a average height of 9.55 ± 0.08 μm (see Figure S3 for a profile measurement of the pillars). The diameters were close to the intended value of 20 μm while the heights were defined by the actual thickness of the cell that was 10 μm. The microstructures appeared smooth and unpitted. Production speed equaled 0.24 mm^3^ h^–1^ which is 2 orders of magnitude greater than the results reported for TPP-DLW-assisted LCN microfabrication (see SI).^21,30^ Even greater speeds can be achieved by arranging microstructures more densely, increasing an in-plane structure size and optimizing blue and UV light intensities and resist composition. A larger array, spanning a ∼5.4 mm^2^ area and consisting of 75 × 75 = 5625 pillars, could be produced in 37.5 s by tiling subarrays of 15 × 15 microcylinders across the cell (Figure 3b).

To confirm the need for the dual-wavelength approach, the same array of 5 × 5 pillars was printed by using only blue images (Figure S4). The microstructures had an increased average diameter of 22.5 ± 0.9 μm, indicating reduced polymerization confinement in the absence of UV-induced inhibition, something that can be particularly undesirable when printing structures with fine or complex features. This was demonstrated by printing flowers: petals of flowers printed with only blue light merged (Figure S5a), while the dual-wavelength approach allowed one to reproduce the desired flower shape more accurately (Figure S5b).

The next step was to generate molecular alignment within the LC photoresist to obtain micropillars with programmed shape changes. This could be readily accomplished in the traditional manner of applying alignment layers on the cell substrates. The polarized optical microscopy (POM) images of pillars printed in a planar cell are shown in Figure 3c(i). The prints appeared bright when the director and polarizer were at 45° with respect to each other and dark when they were parallel, indicating that the planar orientational order was successfully preserved during printing.

To test the thermally induced actuation of the pillars, the structures were heated to 200 °C. As expected, due to the molecular order decrease, the pillars contracted along the director and expanded in the perpendicular direction, forming an ellipsoidal shape when viewed from directly overhead (Figure 3c(ii)). Side profile measurements of a single pillar indicated a height increase. Strains of 27% for expansion and ∼(−26)% for contraction were achieved. These values are greater than results typically reported for LCN microstructures,^21,30,41,42^ and greater than expected for the employed cross-linker concentration. This is most likely related to incomplete monomer conversion, which could be improved through optimization of the resist photoresponse in future studies.

Two other actuation modes could be realized for the same pillars by simply changing the alignment layers to induce homeotropic or π/2-twist director configurations. In contrast to the planar structures, the homeotropically aligned cylinders exhibited a uniform diameter increase (Figure 3d) and a decrease in height upon heating (Figure S6). The π/2-twist pillars became longer and underwent anisotropic expansion/contraction along the long axis, resulting in a truncated cone-like shape when observed from the side (Figure 3e). All of the actuations described were reversible, with the structures returning to their initial shape upon cooling.

**Figure 4:**
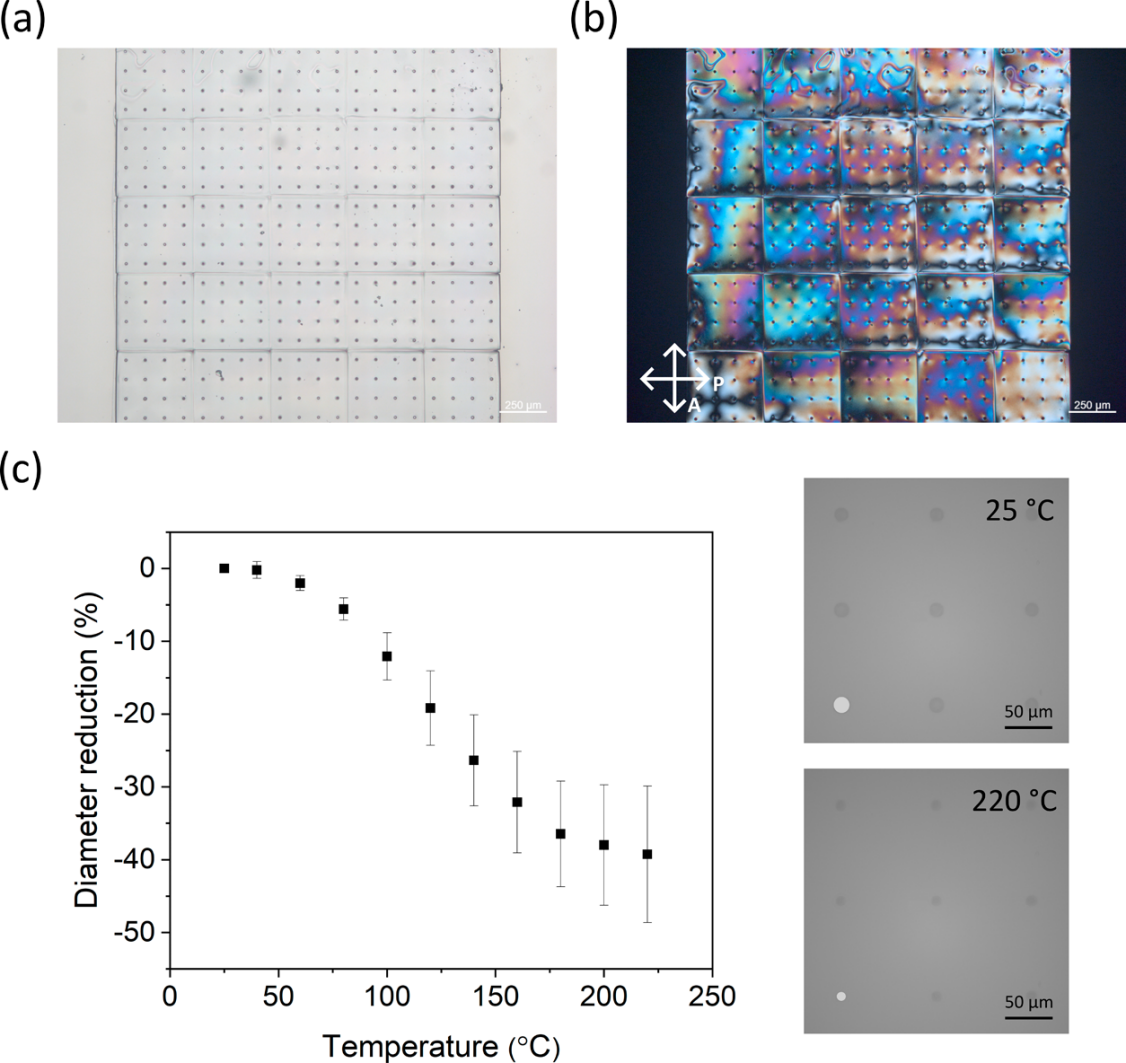
(a) Reflection and (b) POM images of a membrane with tunable pores. (c) Pore size reduction as a function of temperature (left) and reflection images of the pores at 25 and 220 °C (right). The lower left pore in each image is highlighted with a lighter shade of gray.

Finally, DWVML was used to fabricate an alternative 4D LCN microstructure: 25 tiles each comprising 4 × 4 arrays of ∼15 μm diameter holes (400 pores in total) were printed throughout a homeotropic cell to make a surface-bound membrane with tunable pores (Figure 4a). The structure did not appear completely dark through crossed polarizers (Figure 4b) indicating that the alignment was somewhat distorted, likely caused by an observed material flow induced by polymer shrinkage, significant for the large surface-area membrane. Despite this reduced molecular order, the pores contracted upon heating, suggesting the homeotropic alignment was locked in the basal layers, which were printed first. The shape deformations onset at around *T*_g_ = 27 °C when the network enters its more soft, rubbery state, and gradually progressed until saturating at around *T*_NI_ ≈ 210 °C. The shape of the response curve is similar to previous observations.^30^ A maximum of 40% diameter decrease could be achieved corresponding to a 5% strain of the membrane itself (Figure 4c). This strain is smaller than that observed for the pillars, which can be explained by adhesion to the substrate enhanced by the large contact area as well as the buildup of compressive stresses at the pores’ perimeter. By adjustment of the resist composition and the initial pore size, a membrane with pores that completely close upon heating can be potentially prepared.

## Conclusions

Dual-wavelength volumetric microlithography was successfully applied to LCN microfabrication. A photoresist was prepared by using existing LCN chemistry without the need for new molecules. Thousands of well-defined pillars could be produced in seconds over areas as large as ∼5.4 mm^2^. Thanks to in-cell printing, different alignments (planar, homeotropic, twist) could be induced in the traditional way of coating alignment layers to make pillars with programmed actuation modes. The resulting microstructures actuated in the expected manner in response to the temperature, reaching strains of up to 27%. A membrane whose pore size reduced to almost half upon heating was also fabricated, although a certain degree of alignment distortion was observed due to the polymer shrinkage-induced material flow.

More detailed and systematic studies on the DWVML-processing of LCNs will help reveal the full potential as well as limitations of the technology when applied to 4D microfabrication. Optimization of the resist photoresponse and printing process can be performed to further increase the fabrication speed, improve monomer conversion, and prevent alignment distortion occurring during printing large-area micro-objects. Making shapes more complex, including 2.5 and 3D, will be explored in future work. The effect of incorporation of dyes and photothermal, conductive, or magnetic nanoparticles on the photoinitiation and inhibition is interesting to investigate to realize LCN microactuators that are responsive not only to temperature but also to light and electric and magnetic fields. While many questions still need to be answered, DWVML already promises to become a powerful tool for both research and the potential large-scale manufacturing of microstructures and devices based on stimuli-responsive polymer materials.

## Experimental Section

### Materials

LC monomers 4-[[[4-[(1-Oxo-2-propenyl)oxy]butoxy]carbonyl]oxy]benzoic acid 2-methyl-1,4-phenylene ester (LC242) and 4-Methoxybenzoic acid 4-(6-acryloyloxy-hexyloxy)phenyl ester (C6BP) were acquired from BASF and Synthon, respectively. Photoinitiator camphorquinone (CQ) was purchased from ABCR. Co-initiator ethyl 4-(Dimethylamino)benzoate (EDAB) and photoinhibitor 2,2′-Bis(2-chlorophenyl)-4,4′,5,5′-tetraphenyl-1,2′-biimidazole (*o*-Cl-HABI) were obtained from TCI. Chiral dopant benzoic acid, 4-hexyl-,4-[[(1-methylheptyl)oxy]carbonyl]phenyl ester (ZLI-811), was acquired from Merck.

Coatings used for cell preparation included OPTMER AL 1254 from JSR (planar alignment), Sunever 5661 from Nissan Chemical (homeotropic alignment), 3-(trimethoxysilyl)propyl methacrylate (adhesive coating), and trimethoxy(octadecyl)silane (antiadhesive coating) from Sigma-Aldrich.

The developer solution comprised isopropyl alcohol (IPA) from Biosolve, propylene glycol monomethyl ether acetate (PGMEA) from Aldrich, and methyl isobutyl ketone (MIBK) from Acros Organics. Dichloromethane (DCM) and tetrahydrofuran (THF) were purchased from Biosolve.

### Liquid Crystal Photoresist Preparation

The LC photoresist consisted of ∼57.96 wt % C6BP, ∼38.64 wt % LC242, ∼2 wt % CQ, ∼0.4 wt % EDAB, and ∼1 wt % o-Cl-HABI. The components were mixed in DCM for 30 min with the help of a magnetic stirrer, after which the solution was run through a 0.2 μm filter. To evaporate the solvent, the material was kept on a hot plate at 40 °C for 2 h and then left in a fume hood overnight. The resulting photoresist was placed in a vacuum oven at room temperature for 2 days to ensure complete removal of DCM. To produce pillars with twist alignment, a trace amount of the chiral dopant ZLI-811 was added to the mixture.

### DSC Measurements

The phase behavior of the photoresist was studied using a DSC Q2000 (TA Instruments) with aluminum hermetic pans. The DSC measurement was performed on a photoresist mixture without initiating and inhibiting agents to avoid polymerization. The temperature varied between −50 and 150 °C at a rate of 10 °C min^–1^. The corresponding LCN material was also characterized. For that, a polymer film was prepared by placing the resist in the isotropic state between two glass slides, cooling the system to room temperature, and exposing it to 600 mW cm^–2^ light for 2 min followed by post polymerization at 120 °C for 30 min. The DSC measurement was run between −50 and 270 °C at the rate of 5 °C min^–1^ in this case.

### UV–Vis Spectroscopy Measurements

Absorbance spectra were recorded with a UV-3102 PC spectrophotometer (Shimadzu) using 2.5 mM solutions of the resist components in THF. 10 μm thick planar cells filled with C6BP or LC242 were used to characterize the absorbance of the nondiluted monomers.

### FTIR Measurements

The monomer conversion as a function of time was measured with a 670IR FTIR spectrometer (Varian) with a Golden Gate ATR module. The photoresist in the isotropic state was placed on an ATR crystal, covered with a coverslip to form a thin layer, and allowed to cool to room temperature to enter its supercooled nematic phase. A home-built setup composed of 385 and 455 nm LEDs was mounted on top of the ATR crystal with the sample to project blue or/and UV light during the measurements. The conversion was measured by monitoring the height of the acrylate C=C bond peak (1635 cm^–1^).

### Cell Preparation

Cells used for DWVML consisted of a 1 mm glass slide and a 170 μm glass coverslip. The substrates were cleaned by sonication in acetone (20 min) and isopropanol (20 min), followed by a 20-min UV-ozone treatment (Ultra Violet Products, PR-100) to ensure further cleaning and surface activation for spin coating. Once coated with the required materials, the glass slide and coverslip were assembled into a cell with the help of UV-curable glue dispersed with 20 μm beads.

Pillars used for the SEM characterization were printed in cells with an adhesive coating on the bottom substrate and an antiadhesive coating on the top substrate. The adhesive coating consisted of 2 wt % of 3-(trimethoxysilyl)propyl methacrylate and 98 wt % of a solution of 95 wt % water, 5 wt % ethanol, and acetic acid (pH = 4.5). The antiadhesive coating comprised 2 wt % of trimethoxy(octadecyl)silane and 98 wt % of the same solution. Once prepared, both mixtures were shaken for 30 min and then filtered with a 0.2 μm filter. Spin coating of the glass with adhesive/antiadhesive layers was done with a Karl Suss CT 62 spin coater set to 15 s at 3000 rpm with an acceleration of 100 rpm s^–1^. Coated glass slides were baked at 65 °C for at least 10 min and then rinsed with ethanol.

All of the other microstructures were printed in cells with alignment layers. Depending on the desired director configuration, OPTMER AL 1254 (planar alignment) or Sunever 5661 (homeotropic alignment) was spin-coated in two steps: (1) 800 rpm for 5 s; (2) 5000 rpm for 40 s, both steps preceded by a 500 rpm s^–1^ acceleration. After spin coating, substrates were baked first at 100 °C for 20 min and then at 180 °C for 2 h. Glass plates coated with OPTMER AL 1254 were uniaxially rubbed by means of a velvet cloth and arranged in a parallel fashion to achieve the planar alignment and in an orthogonal fashion to achieve the π/2-twist director configuration.

### DWVML-Printing

Cells were filled with the LC photoresist at 80 °C (in the isotropic phase) and then slowly cooled to room temperature to minimize defects. The microstructures were printed layer by layer from the bottom to the top cell substrate. The translation stage moved continuously at a speed of 14.4 nm ms^–1^. The projected image changed each 5.8 ms. The effective layer thickness was defined by multiplying the stage speed by the time delay between two consecutive blue images, i.e., 11.6 ms, and equaled 167 nm. It must be noted that polymerization is not confined to the 167 nm layer of the material but spreads to some extent along the z direction, resulting in a gradual transition between layers. Blue and UV light intensities of *I*_blue_ = 29.4 mW cm^–2^ and *I*_UV_ = 304.1 mW cm^–2^ and an overall printing time of 1.2 s were employed. The blue and UV images used to print the reported microstructures are given in Figure S7. The 75 × 75 array and membrane were produced by printing several subarrays or tiles, respectively, next to each other. Once printing was finished, the cell was placed in a developer solution of PGMEA, MIBK, and IPA for 15 min to remove nonpolymerized material. Finally, the cell was opened and rinsed first with the developer solution and then with ethanol to remove the leftover photoresist. The printed microstructures remained attached to the substrate with the adhesive layer or to one of the two substrates in the case of cells with alignment layers.

### Microstructures Characterization

Electron micrographs were acquired with JEOL JSM-IT100 and Quanta FEG 3D scanning electron microscopes in secondary electron mode with a 5 kV acceleration voltage. Reflection and POM images were recorded with a Leica DM2700 M polarized optical microscope. Images characterizing actuation of the pillars were acquired with a Sensofar optical profilometer in reflection microscopy mode. The temperature of the LCN actuators was controlled by a THMS 600 hot stage (Linkam).

## Supporting Information Available

DSC measurements of the LC photoresist and corresponding LCN; UV-induced initiator-less polymerization tests; profile measurement of a 5 × 5 micropillar array; comparison of DWVML and TPP-DLW LCN fabrication speeds; comparison of single-wavelength and dual-wavelength approaches; profile measurement showing actuation of the homeotropic micropillar; blue and UV images used for DWVML-printing (PDF)
