# Supplementary material for: Dual-Wavelength Volumetric Microlithography for Rapid Production of 4D Microstructures
Source: ACS Appl Mater Interfaces. 2024 Apr 22;16(17):22696–703. doi: 10.1021/acsami.4c01883 (PMC11071039; doi:10.1021/acsami.4c01883)
Supplement: Supplementary file 1 — am4c01883_si_001.pdf [file am4c01883_si_001.pdf]

Supporting Information

Dual-Wavelength Volumetric Micro-Lithography for Rapid Production of 4D Microstructures

*Alexandra Gruzdenko†, ‡, §, Dirk J. Mulder‖, Albert P. H. J. Schenning†, ‡, §, Jaap M. J. den Toonder⊥, ‡. §, and Michael G. Debije†, ‡**

† Stimuli-responsive Functional Materials and Devices, Department of Chemical Engineering and Chemistry, Eindhoven University of Technology, P.O. Box 513, 5600 MB Eindhoven, The Netherlands.

⊥ Microsystems, Department of Mechanical Engineering, Eindhoven University of Technology, P.O. Box 513, 5600 MB Eindhoven, The Netherlands

‡ Interactive Polymer Materials (IPM), Eindhoven University of Technology (TU/e), Groene Loper 3, 5612 AE Eindhoven, The Netherlands

§ Institute for Complex Molecular Systems, Eindhoven University of Technology, Den Dolech 2, 5600 MB Eindhoven, The Netherlands

‖ Photosynthetic, De Boelelaan 1085, 1081HV Amsterdam, The Netherlands

*Corresponding author's email: m.g.debije@tue.nl

Phase Behaviour of The Liquid Crystal Photoresist and Corresponding LCN Materia

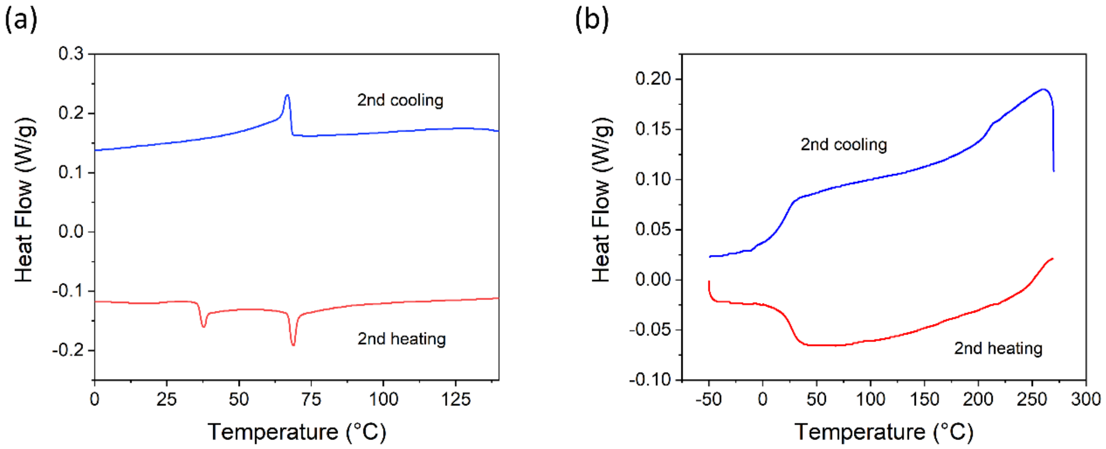

Figure S1. (a) DSC thermogram of a C6BP-LC242 mixture, without the initiating and inhibiting agents to avoid polymerization, with the same weight ratio as used for the LC photoresist. (b) DSC thermogram of LCN material prepared from the photoresist by photopolymerization.

UV-induced Initiator-less Polymerization

UV-Vis measurements performed on non-diluted C6BP and LC242 did not indicate significant absorption at 385 nm (**Figure S2a**). Moreover, the pure monomers did not polymerize upon 1 min exposure to 304.1 mW cm^{-2} UV light according to FTIR measurements (**Figure S2b**). Therefore, any UV-induced polymerization is likely related to the CQ absorption tail.

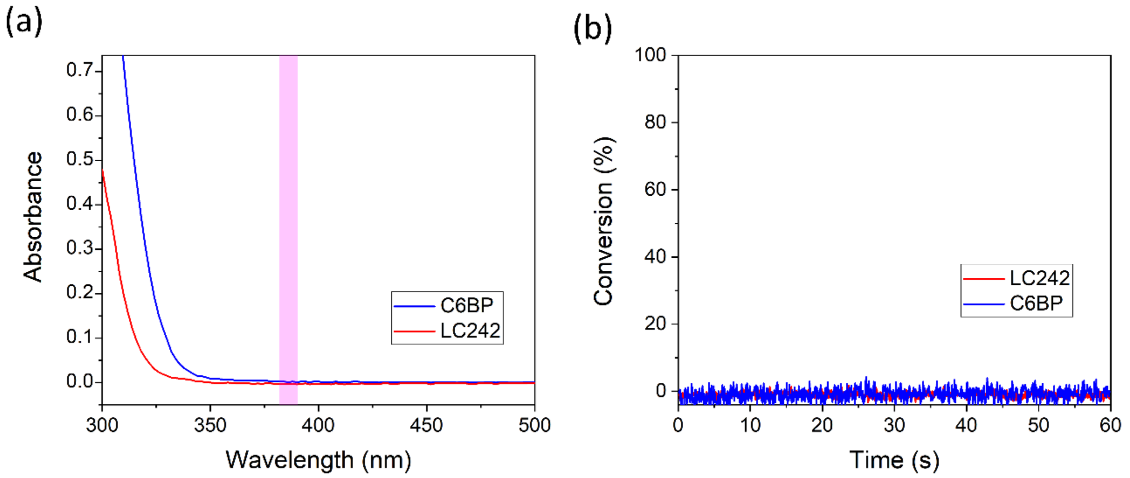

Figure S2. (a) Absorbance spectra of bulk C6BP and LC242. (b) Acryalte conversion as a function of time measured for C6BP and LC242 continuously illuminated with 304.1 mW cm^{-2} UV light.

Profile Measurement of Pillars of The 5×5 Micropillar Array

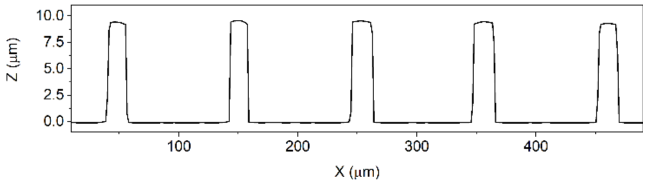

Figure S3. Profile measurement of the first (front) row of the pillar array shown in Figure 3a.

Comparison of DWVML and TPP-DLW LCN Fabrication Speeds

In previous work,[1] an LCN hexagonal plate with a ~2.5 µm height and ~20 µm diagonal was TPP-DLW-printed in 1.25 s corresponding to a 0.002 mm^3 h^{-1} production speed. In other work,[2] a typical voxel size of 2×2×2 μm^3 and a printing speed of 90 µm s^{-1} were employed for TPP-DLW resulting in an estimated rate of 0.001 mm^3 h^{-1}. The 5×5 array of pillars with a ~10 µm height and ~20 µm diameter described in this work was DWVML-printed in 1.2 s resulting in a 0.24 mm^3 h^{-1} production speed.

Single VS Dual-Wavelength Approaches

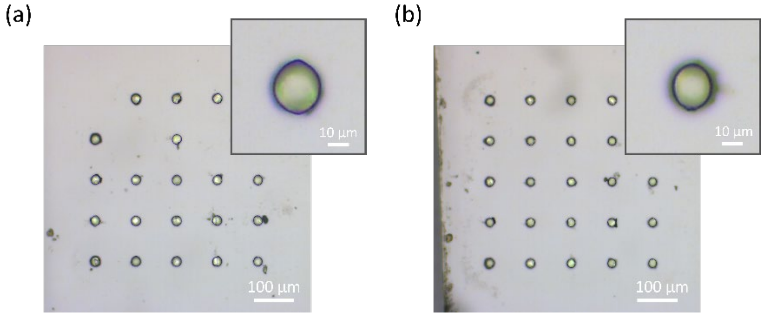

Figure S4. Reflection images of 5×5 pillar arrays printed (in 1.2 s) using only blue (a) and both blue and UV (b) images. The target pillar diameter was 20 µm; the actual average diameter was 22.5±0.9 µm for pillars printed with the single wavelength approach and 19.0 ± 0.8 µm for pillars printed with the dual-wavelength approach. The overall duration of exposure to blue light was 0.6 s in both cases. Some of the pillars of the array shown in image (a) detached from the substrate during the washing step.

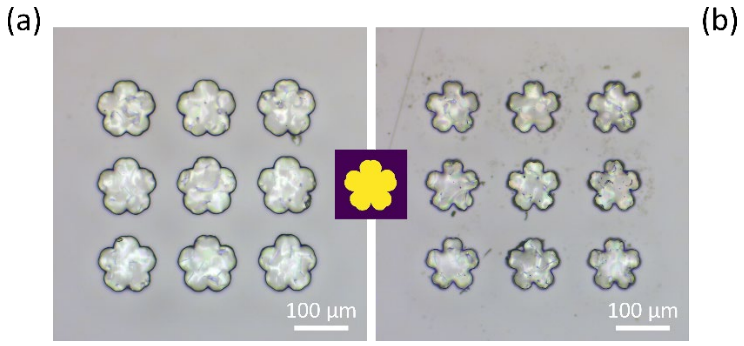

Figure S5. Reflection images of 3×3 arrays of flowers printed (in 1.2 s) using only blue (a) and both blue and UV images. The inset in the centre shows the target flower shape. The overall duration of exposure to blue light was 0.6 s in both cases.

Homeotropic-pillar Actuation

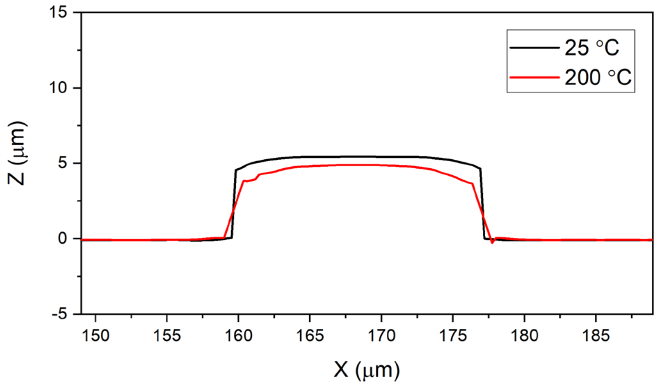

Figure S6. Homeotropic pillar profiles measured at 25 °C (black) and 200 °C (red).

Blue and UV Images Used For DWVML-Printing

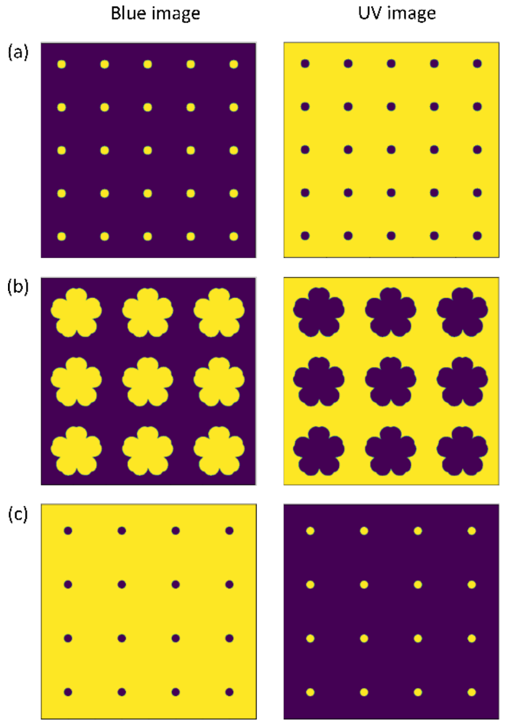

Figure S7. Blue and UV images used to print the 5×5 pillar array (a), 3×3 flower array (b), and membrane (c). Yellow areas correspond to regions where blue/UV light was projected; in the purple areas, there was no illumination. Since the structures did not vary in the z direction, the same blue and UV images were used for all layers. One micromirror created a 0.6 µm × 0.6 µm pixel in the final image projected by the objective.

References

(1) del Pozo, M.; Delaney, C.; Pilz da Cunha, M.; Debije, M. G.; Florea, L.; Schenning, A. P. H. J. Temperature-Responsive 4D Liquid Crystal Microactuators Fabricated by Direct Laser Writing by Two-Photon Polymerization. *Small Struct* **2022**, *3* (2), 2100158. https://doi.org/10.1002/sstr.202100158.

(2) Nocentini, S.; Martella, D.; Parmeggiani, C.; Wiersma, D. Photoresist Design for Elastomeric Light Tunable Photonic Devices. *Materials* **2016**, *9* (7), 525. https://doi.org/10.3390/ma9070525.
